# Siblings With Berardinelli-Seip Congenital Lipodystrophy: Clinical Insights and Challenges

**DOI:** 10.7759/cureus.75434

**Published:** 2024-12-09

**Authors:** Sri Meghana Kankipati, Surbhi Dumra, Swati Thareja, Lyluma Ishfaq, Mah N Zargar, Arghadip Das, Sreya Kongala, Salma Younas

**Affiliations:** 1 Medicine and Surgery, Andhra Medical College, Visakhapatnam, IND; 2 Medicine, Employees' State Insurance Corporation (ESIC) Medical College and Hospital, Faridabad, IND; 3 Internal Medicine, The Hans Foundation, Delhi, IND; 4 Medicine, Central Michigan University College of Medicine, Saginaw, USA; 5 Medicine, Fatima Jinnah Medical University, Lahore, PAK; 6 Internal Medicine, Nilratan Sircar Medical College and Hospital, Kolkata, IND; 7 Internal Medicine, Osmania Medical College, Hyderabad, IND; 8 Pharmacy, Punjab University College of Pharmacy, Lahore, PAK

**Keywords:** berardinelli-seip syndrome, congenital lipodystrophy, genetic mutation, hepatic steatosis, hyperglycemia, insulin resistance, lipid metabolism disorder, multisystemic complications, pediatric case series, skeletal muscle hypertrophy

## Abstract

Berardinelli-Seip congenital lipodystrophy (BSCL), also known as congenital generalized lipodystrophy (CGL), is an exceptionally rare autosomal recessive disorder marked by a significant deficiency of adipose tissue throughout the body. This lack of adipose tissue, normally found beneath the skin and between internal organs, leads to impaired adipocyte formation and fat storage, causing lipids to accumulate in atypical tissues such as muscles and the liver. The extent of adipose tissue loss directly influences the severity of symptoms, which can include a muscular appearance, increased appetite, bone cysts, marrow fat depletion, acromegalic features, severe insulin resistance, skeletal muscle hypertrophy, hypertrophic cardiomyopathy, hepatic steatosis, hepatomegaly, cirrhosis, and intellectual disability.

We present a case series of two siblings with BSCL: a nine-year-old boy and his seven-year-old sister, each with unique manifestations of the disorder. The older sibling presented with high-grade fever and right ankle pain, possibly indicative of a calcified deposit, alongside complications such as hyperglycemia (managed without insulin) and moderate pulmonary arterial hypertension (PAH) with tricuspid regurgitation (TR). The younger sibling displayed similar metabolic and cardiovascular complications, including hepatomegaly and early signs of cardiac involvement. Both cases required comprehensive evaluations, revealing anemia, thrombocytopenia, elevated leukocyte count, and high C-reactive protein (CRP) levels. The children were managed with high-potency antibiotics, leading to a marked improvement in their overall conditions.

These cases demonstrate the broad spectrum of clinical manifestations associated with BSCL and highlight the importance of a multidisciplinary approach for effective management. Although limited by the small sample size, this case series shows the importance of a multidisciplinary approach in addressing the complex and overlapping symptoms of BSCL, which often mimic more common conditions. Increased awareness among healthcare providers is crucial for ensuring timely diagnosis and appropriate intervention, particularly in pediatric patients.

## Introduction

The recent Orphanet inventory estimates that over 7,000 rare diseases exist worldwide, with 60%-80% attributed to genetic disorders, and around 70% affecting children in the early years of development [1,2]. Among these, congenital generalized lipodystrophy (CGL) is a rare autosomal recessive disorder that demands recognition in the healthcare sector due to its severe, early-onset complications and association with high morbidity and mortality rates [2]. Often categorized within a broader group of lipodystrophies, CGL is defined by an extreme scarcity of adipose tissue. However, lipodystrophy encompasses a range of disorders that disrupt adipose tissue homeostasis, resulting in abnormal distribution of fat throughout the body [3].

In CGL, the body's capacity to store fat in adipocytes is severely impaired, leading to lipid accumulation in atypical sites such as muscle, liver, heart, and vessel walls [2]. Clinically, this disorder is characterized by developmental delays, coarse acromegaloid facies, and metabolic derangements, such as diabetes mellitus with insulin resistance, often manifesting as acanthosis nigricans. Additional features include early-onset hypertrophic cardiomyopathy, hepatomegaly, skeletal muscle hypertrophy, and hormonal disturbances that may cause infertility in both men and women [3,4]. These complications underscore the multisystemic impact of CGL, making its early detection and management critical.

Genetically, CGL is most commonly linked to mutations in the BSCL2 and AGPAT2 genes, which play essential roles in lipid metabolism and adipocyte development [4]. Mutations in BSCL2, which encodes the protein seipin, and AGPAT2, which encodes lysophosphatidic acid acyltransferase B, lead to biochemical disruptions such as hypertriglyceridemia, hypoadiponectemia, and hyperglycemia [4,5]. Molecular studies utilizing polymerase chain reaction (PCR) have shown increased endoplasmic reticulum stress and lipid peroxidation associated with these mutations, further illustrating the disease’s complex pathophysiology [5].

While treatment options for lipodystrophy have expanded beyond lifestyle and metabolic management to include FDA-approved metreptin therapy, the drug remains costly and shows limited efficacy, highlighting the ongoing need for alternative treatments [6]. The annual cost of metreleptin therapy is approximately $1,139,730 per patient, depending on vial size and dosage. While metreleptin is effective in improving key metabolic parameters, its efficacy is variable [7]. For example, a study by Oral et al. (2019) demonstrated reductions in glycated hemoglobin (HbA1c) by 0.6% and fasting triglycerides by 20.8% in the overall population after 12 months of treatment [8]. Given the extreme rarity of CGL, affecting approximately one in 10,000,000 newborns, and its clinical overlap with common conditions like malnutrition, diagnosis is often delayed, adversely impacting patient prognosis [2].

This case series aims to provide the unique presentation of CGL in two siblings and the challenges in diagnosis and management due to the disorder’s rarity and complex symptomatology. Through these cases, we discuss the necessity of a multidisciplinary approach to address the diverse complications of CGL and to improve patient outcomes.

## Case presentation

Case one

History

The patient was a nine-year-old male who was hospitalized for approximately three weeks. He presented with a high-grade fever lasting three days and was referred from a private hospital with thrombocytopenia.

The patient’s primary complaint was a high-grade fever that began three days before his admission. The fever was intermittent and not associated with chills, rigors, vomiting, cough, shortness of breath, or other respiratory symptoms. The parents reported no episodes of loose stools, abdominal distension, or urinary complaints such as hematuria or burning micturition. There were also no seizures, rashes, or bleeding manifestations during this period.

His medical history was significant for CGL, a rare disorder associated with insulin resistance. He has not been hospitalized previously, nor has he required any blood transfusions. There was no known contact history with tuberculosis.

The patient was born at term via cesarean section with a birth weight of 3.7 kg. His postnatal period was uneventful, and he achieved developmental milestones appropriate for his age. He has been immunized according to the recommended schedule.

In terms of family history, the patient’s parents are consanguineously married, but there is no family history of congenital heart disease (CHD), kidney disease, epilepsy, or tuberculosis.

Clinical Examination

On general examination, the child was found to be conscious and coherent, with vital signs as follows: heart rate of 104 beats per minute, respiratory rate of 22 breaths per minute, oxygen saturation (SpO_2_) of 95% on room air, and blood pressure of 108/60 mmHg. His random blood glucose was measured at 119 mg/dL.

Anthropometric measurements revealed a weight of 33 kg and a height of 137 cm. Notable physical features included acanthosis nigricans in the neck and axilla, a general lack of subcutaneous fat, and a muscular build. His facial features appeared coarse, with a prominent supraorbital region and thick lips.

Systemic examination findings were unremarkable. In the cardiovascular system, both heart sounds (S1 and S2) were normal, with no murmurs detected. His respiratory system examination revealed bilateral normal vesicular breath sounds, with no added sounds. The central nervous system examination showed bilaterally reactive pupils, normal tone, and no focal neurological deficits. The abdominal examination indicated a normal shape, with a soft abdomen and no organomegaly or abnormalities upon palpation or percussion.

**Figure 1:**
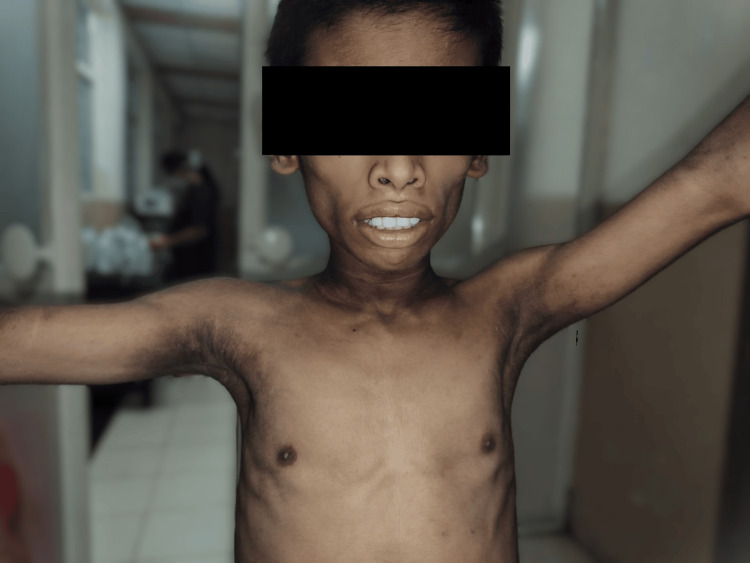
Clinical appearance of Berardinelli-Seip congenital lipodystrophy (BSCL) in the older sibling with muscular build and acanthosis nigricans

Figure 1 shows reduced subcutaneous fat, muscular build, and skin features (acanthosis nigricans), which are characteristic of BSCL.

Laboratory Investigations

The patient’s laboratory investigations were monitored across multiple days, providing insights into various parameters. His complete blood count (CBC) revealed variations in hemoglobin levels and fluctuating platelet counts, indicating an initial elevated count followed by thrombocytopenia. White blood cell counts showed a range suggestive of immune response, with specific trends in neutrophils and lymphocytes. Biochemical analyses identified mild abnormalities in electrolyte levels and liver function, while kidney function remained stable. Additional tests covered inflammatory markers, glucose regulation, and lipid profile, further detailing his metabolic state.

Table 1 summarizes the laboratory findings.

**Table 1:** Laboratory findings of Case one

Test category	Parameter	Results	Normal range
Complete blood count	Hemoglobin (Hb)	7.5 - 10.5 g/dL	11.5 - 15.5 g/dL
	Packed cell volume (PCV)	27.3% - 31%	35% - 45%
	Platelet count	Initial: 491,000; Later: 92,000 (thrombocytopenia)	150,000 - 450,000/μL
	White blood cell (WBC) count	10,400 - 141,800	4,500 - 13,500/μL
	Differential count	Neutrophilia, lymphopenia	Neutrophils: 40% - 70%; Lymphocytes: 20% - 40%
Biochemical tests	Sodium	132 mmol/L	135 - 145 mmol/L
	Potassium	4.2 mmol/L	3.5 - 5.0 mmol/L
	Chloride	96 mmol/L	98 - 107 mmol/L
Liver function tests	Total bilirubin (TBIL)	1.9 mg/dL	0.1 - 1.2 mg/dL
	Alkaline phosphatase (ALP)	162 U/L	100 - 350 U/L (age-dependent)
	Serum glutamic-oxaloacetic transaminase (SGOT)	22 U/L	5 - 40 U/L
	Serum glutamic pyruvic transaminase (SGPT)	26 U/L	5 - 35 U/L
Renal function tests	Urea	35 mg/dL	7 - 20 mg/dL
	Creatinine	0.8 mg/dL	0.5 - 1.0 mg/dL
Inflammatory markers	C-reactive protein (CRP)	Positive	Negative
	Dengue serology	Negative	Negative
Glycemic control	Glycated hemoglobin (HbA1C)	4.7% (suggesting good control)	4% - 5.6%
Lipid profile	Total cholesterol	61 mg/dL	<170 mg/dL
	Triglycerides (TG)	131 mg/dL	<90 mg/dL (fasting)
	High-density lipoprotein (HDL)	13 mg/dL	>45 mg/dL
	Low-density lipoprotein (LDL)	22 mg/dL	<110 mg/dL
	Very-low-density lipoprotein (VLDL)	26 mg/dL	<30 mg/dL
Oral glucose tolerance test (OGTT)	Fasting glucose	78 mg/dL	70 - 99 mg/dL
	Post-prandial glucose	99 mg/dL	<140 mg/dL

These laboratory findings provide a comprehensive overview of the patient's health, highlighting mild electrolyte imbalances, stable but closely monitored renal function, and controlled blood glucose levels. The lipid profile reflects low HDL levels, consistent with his diagnosis of lipodystrophy, while the oral glucose tolerance test (OGTT) results show no signs of hyperglycemia at this stage.

Imaging Studies

Ultrasound imaging of the abdomen indicated Grade I fatty liver and moderate splenomegaly. A 2D echocardiogram (echo) revealed a normal left ventricular (LV) size with good systolic function and an ejection fraction of 66%. However, trivial mitral regurgitation (MR), mild aortic stenosis (AS), moderate tricuspid regurgitation (TR), and moderate pulmonary arterial hypertension (PAH) were noted.

An X-ray of the right ankle was conducted due to reported pain and swelling in that area, revealing a radiopaque area suggestive of calcification near the medial malleolus. A FibroScan of the liver indicated F4 stage fibrosis, which is likely related to the patient’s lipodystrophy and insulin resistance.

Management and Treatment

The patient was treated for severe infection, including pneumonia and pneumococcal pneumonia, using broad-spectrum antibiotics: cefotaxime, piperacillin + tazobactam, meropenem, and vancomycin. Renal function improved with treatment, though thrombocytopenia persisted. Pain in the right medial malleolus prompted an orthopedic examination, and an X-ray confirmed calcification, leading to a diagnosis of ankle exostosis.

At discharge, the treatment plan included oral supplements such as B-complex vitamins, folic acid (5 mg daily), and iron (ferrous sulfate 100 mg daily). Further investigations were planned, including an MRI of the ankle. A follow-up appointment in the pediatric outpatient department (OPD) was scheduled in two weeks. Dietary recommendations were provided by endocrinology, and further testing was advised, including a 75 gm OGTT and a FibroScan to assess liver fibrosis after six months in Stage F4 fibrosis. Orthopedic follow-up was recommended to exclude gout, with uric acid levels at 3.3 mg/dL.

Case two

History

A seven-year-old female, the younger sibling of Case one, was referred with symptoms consistent with CGL, a rare genetic disorder. Like her brother, she exhibits characteristic physical and metabolic symptoms associated with CGL, including a lack of subcutaneous fat and unique facial features. She was born full-term via lower segment cesarean section with a birth weight of approximately 3.3 kg. Her early neonatal history included no significant complications, and her developmental milestones had been age-appropriate. She was following a mixed diet, and her immunizations were up-to-date as per the recommended pediatric schedule.

Family and Genetic Background

The patient comes from a consanguineous family background, with the parents being first-degree relatives, which increases the likelihood of inherited genetic conditions. No other significant family history was reported for CHD, chronic kidney disease (CKD), epilepsy, or tuberculosis. While genetic testing had been conducted previously, the family does not possess the report, which could provide further insight into the inheritance pattern and specifics of the genetic mutation responsible for CGL in this family.

Clinical Findings and Physical Examination

On examination, the patient displayed hallmark signs of CGL. She had pronounced acanthosis nigricans around the neck and axilla, reflecting metabolic disturbances common in lipodystrophy syndromes. She exhibited a severe reduction in subcutaneous fat, giving her a lean, muscular appearance. Her facial features were distinct, with a prominent supraorbital ridge, thick lips, hollow cheeks, and a large nose, traits commonly associated with CGL. 

**Figure 2:**
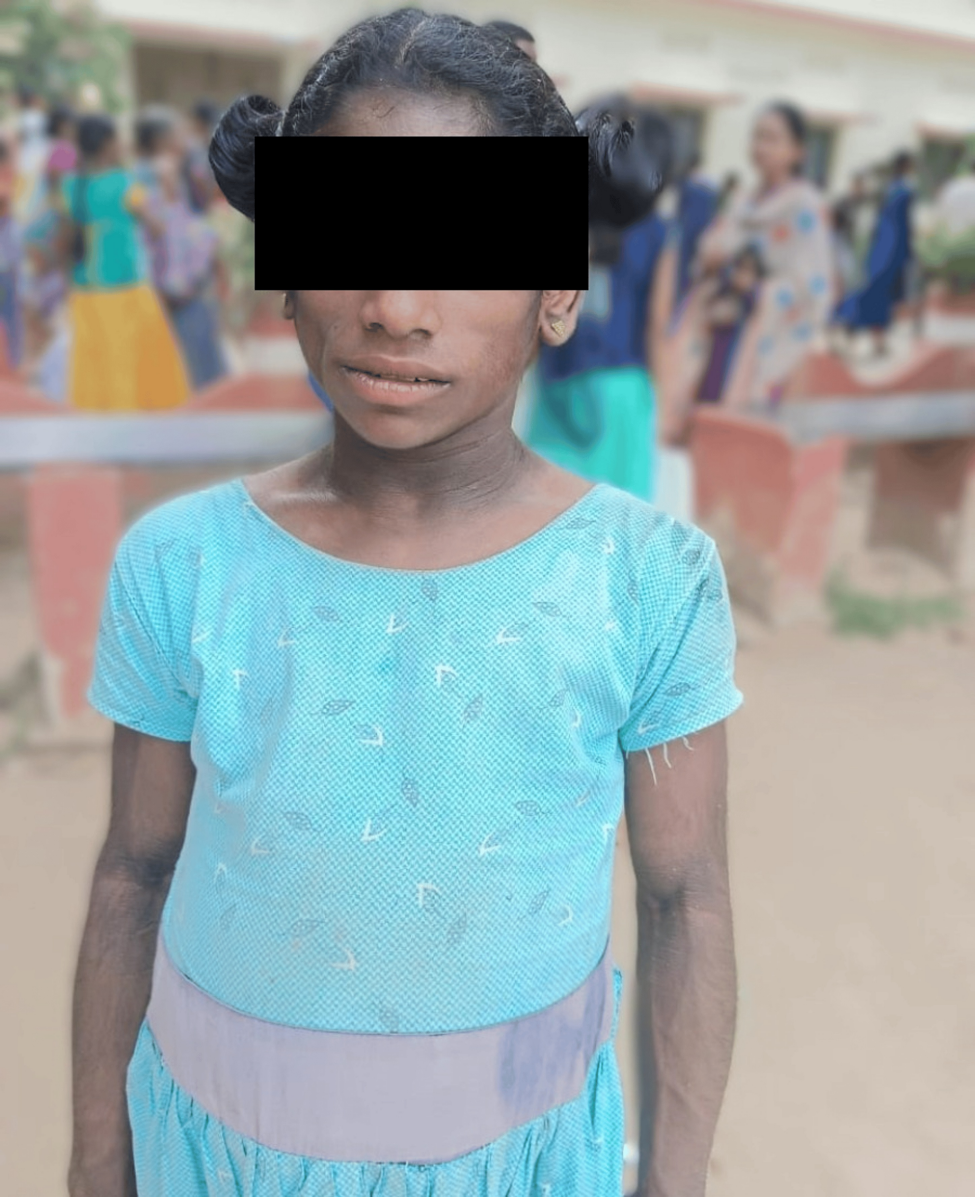
Distinct facial features and lack of subcutaneous fat in the younger sibling with Berardinelli-Seip congenital lipodystrophy (BSCL)

Figure 2 shows the distinct facial characteristics and severe reduction in subcutaneous fat seen in the younger sibling, typical of CGL.

Vital signs on admission were within normal limits, with a heart rate of 99 beats per minute, a respiratory rate of 24 breaths per minute, SpO₂ of 98%, and normal blood pressure readings. There were no signs of pallor or edema, and systemic examinations of the cardiovascular, respiratory, and central nervous systems showed no immediate abnormalities.

Laboratory and Imaging Findings

Laboratory workup indicated mild anemia, with lower-than-average hemoglobin levels. Platelet counts were within normal ranges, and total leukocyte count was mildly elevated, indicating an inflammatory response. The lipid profile revealed slight dyslipidemia, with elevated triglyceride and cholesterol levels, which are consistent with metabolic issues related to lipodystrophy.

Table 2 summarizes the laboratory findings.

**Table 2:** Laboratory findings of Case two

Test	Value	Normal Range	Interpretation
Hemoglobin	10.5 g/dL	12-15 g/dL	Mild anemia
Platelet count	250,000 /µL	150,000-450,000 /µL	Within normal range
Total leukocyte count	11,000 /µL	4,000-10,000 /µL	Mildly elevated, indicating an inflammatory response
Triglycerides	180 mg/dL	<150 mg/dL	Elevated, consistent with dyslipidemia
Cholesterol	220 mg/dL	<200 mg/dL	Elevated, consistent with dyslipidemia

Abdominal ultrasound findings showed a Grade 1 fatty liver and mild splenomegaly. There was also evidence of mild renal dilation bilaterally, indicating potential renal involvement, a finding that warrants close monitoring in patients with CGL.

Orthopedic Evaluation

The patient reported pain and swelling in the area around the right ankle’s medial malleolus. An orthopedic consult and X-ray imaging were obtained, revealing radiopaque calcifications just below the medial malleolus, which could suggest metabolic bone changes commonly seen in CGL. This finding raised concerns for potential orthopedic complications and required further imaging and follow-up to monitor progression.

Cardiac Evaluation

A 2D echocardiogram was performed, revealing situs solitus with levocardia, mild MR, mild AS, moderate TR, and calcification of the aortic valve leaflets. These findings reflect early cardiac involvement, a known complication in congenital lipodystrophy, and indicate the need for regular cardiac monitoring.

Treatment and Hospital Course

During her hospital stay, the patient received broad-spectrum intravenous antibiotics to manage and prevent infection due to her immunocompromised state. The antibiotics included cefotaxime for six days, piperacillin-tazobactam for 10 days, meropenem for five days, and vancomycin for five days. She also received nutritional supplements, including B-complex vitamins, folic acid, and iron, to address her underlying nutritional deficiencies and support her general health.

Discharge Plan and Follow-Up

At discharge, the patient was advised to follow dietary recommendations provided by the endocrinology team to support her metabolic health. She was scheduled for a follow-up MRI of the right ankle to further assess the observed calcifications and rule out other orthopedic complications. A FibroScan was recommended for liver fibrosis assessment, with a follow-up planned in six months to monitor any progression of liver involvement. She was referred to the pediatric OPD for review in two weeks, with a follow-up plan that included regular liver and kidney function monitoring and periodic cardiac assessments to address her mild heart valve issues.

## Discussion

Berardinelli-Seip congenital lipodystrophy, a subtype of CGL, is an autosomal recessive disorder characterized by a near-total absence of adipose tissue. With only 200-300 cases reported worldwide, BSCL remains exceedingly rare, with higher prevalence noted among specific ethnic groups, such as Latin American and Arab populations [9,10]. Diagnosis is typically based on meeting established clinical criteria, though genetic confirmation through AGPAT2 or BSCL2 variants is considered definitive. However, in resource-limited settings, where genetic testing may be inaccessible, clinical criteria remain essential for diagnosis [11].

Congenital generalized lipodystrophy is a rare autosomal recessive disorder with four main subtypes, distinguished by the specific gene mutation causing the condition. Congenital generalized lipodystrophy Type 1, caused by mutations in the AGPAT2 gene, is characterized by a near-complete absence of metabolically active adipose tissue from birth, with residual fat limited to certain mechanical fat depots such as the palms, soles, and orbital regions [12]. Congenital generalized lipodystrophy Type 2, caused by BSCL2 mutations, is typically more severe, involving a complete absence of mechanical and metabolically active fat. It often presents with intellectual impairment, hypertrophic cardiomyopathy, and skeletal anomalies [13,14]. Congenital generalized lipodystrophy Type 3, associated with mutations in CAV1, exhibits variable clinical presentations, including mild lipodystrophy and less severe metabolic complications [15]. Congenital generalized lipodystrophy Type 4, caused by CAVIN1 mutations, is marked by atypical fat distribution, a milder metabolic phenotype, and cardiomyopathy [16].

In this case series, both siblings presented with typical BSCL features, including acanthosis nigricans, a muscular build, and distinct facial characteristics, supporting the clinical diagnosis of BSCL based on major diagnostic criteria [17]. Their metabolic profile, with insulin resistance and dyslipidemia, further aligns with typical presentations of BSCL, although genetic testing was unavailable in this setting.

An unusual finding in these cases was the orthopedic complication of ankle exostosis, noted in the older sibling, which manifested as pain and swelling around the medial malleolus. Radiological imaging and serum uric acid levels confirmed the diagnosis, highlighting an atypical manifestation not commonly documented in BSCL, which adds complexity to the clinical picture [18]. Cardiovascular findings also deviated from classic BSCL presentations, as the 2D echocardiogram revealed mild mitral regurgitation, mild aortic stenosis, moderate tricuspid regurgitation, and PAH rather than the expected hypertrophic cardiomyopathy. These findings underscore the variability in cardiovascular involvement among BSCL patients, suggesting the need for individualized cardiac assessments and tailored management strategies [19].

The study's limitations include the small sample size of only two siblings, which restricts generalizability to other patients with BSCL. The rarity of BSCL and its heterogeneous manifestations pose challenges for establishing standardized diagnostic and management protocols. Lack of genetic testing to confirm specific mutations and limited long-term follow-up constrain the ability to draw comprehensive conclusions about disease progression and outcomes.

The complexity of BSCL is reflected in the broad spectrum of clinical manifestations and associated complications, emphasizing the importance of a multidisciplinary approach. The presence of orthopedic and cardiovascular anomalies in these cases shows the necessity for individualized management to address diverse health challenges. In settings where genetic testing is not readily available, reliance on clinical diagnostic criteria and careful monitoring remains critical for patient care.

Future research should aim to enhance understanding of the genetic pathways in BSCL to improve diagnostic and therapeutic options. Further exploration into the variability in cardiovascular manifestations among BSCL patients may also provide insights into the development of complications and inform treatment protocols. Guidelines for the diagnosis and management of lipodystrophy syndromes, as outlined by Brown et al. (2016), recommend comprehensive clinical evaluation supported by imaging and laboratory findings when molecular testing cannot be accessed [20]. Developing standardized treatment guidelines that incorporate flexible diagnostic approaches and comprehensive management strategies is essential to improve outcomes for individuals affected by this rare condition.

## Conclusions

Managing BSCL is challenging due to its rarity, diverse clinical presentations, and variable treatment responses. Early diagnosis and comprehensive metabolic and endocrine assessments are crucial for optimal patient care. Increasing awareness of BSCL can improve early detection and management. Ongoing research into its genetic mechanisms is key to developing targeted therapies, while standardized guidelines can enhance clinical outcomes for affected individuals.
